# Multigated Photochromic Materials: Mechanism, Progress, and Application

**DOI:** 10.34133/research.1153

**Published:** 2026-03-04

**Authors:** Zihe Song, Jimeng Liao, Fei Hao, Mingyao Shen, Jiamin Wu, Tao Yu

**Affiliations:** ^1^ State Key Laboratory of Flexible Electronics (LoFE) and Institute of Flexible Electronics (IFE), MIIT Key Laboratory of Flexible Electronics, Shaanxi Key Laboratory of Flexible Electronics, Northwestern Polytechnical University, 127 West Youyi Road, Xi’an 710072, China.; ^2^ Key Laboratory of Flexible Electronics of Zhejiang Province, Ningbo Institute of Northwestern Polytechnical University, Ningbo 315103, China.; ^3^ Shandong Provincial Key Laboratory of Molecular Engineering Qilu University of Technology–Shandong Academy of Science, Ji’nan, China.

## Abstract

Photoresponsive materials offer unique opportunities for advanced technologies by enabling their precise, noncontact remote control and converting light energy into well-defined changes in structure and function. Among them, multigated photochromic systems have emerged as a transformative frontier, overcoming constraints of conventional single-stimulus systems. Here, “gated” refers to the deliberate use of an additional external input (e.g., pH, voltage, mechanical force, or temperature) to regulate or modulate the photoisomerization process. Such multi-input control enables sophisticated, programmable behaviors, including Boolean logic operations, environmental adaptation, and high-security information encryption, thereby marked expanding their application potential. In this review, we present a comprehensive and systematic analysis of multigated photochromic materials. We first introduce a unified design strategy and classification framework based on gating mechanisms. The core sections critically evaluate recent advances in proton-, electro-, mechano-, thermal-, and wavelength-gated systems, with particular emphasis on the underlying principles that connect molecular design to tailored performance. Furthermore, we discuss emerging and unconventional gating modes, including ion-, liquid-, gas-, and intensity-gated photochromism. Finally, we present a comparative analysis of all gating modalities, identify persistent conceptual and practical challenges, and outline future directions toward intelligent, adaptive material platforms. This review aims to establish a foundational framework that guides the rational design of next-generation multigated photochromic materials for applications in sensing, anticounterfeiting, information technology, and adaptive devices.

## Introduction

Smart materials are defined as a category of materials capable of sensing external stimuli and actively responding to them, resulting in controllable alterations in their properties or functionalities. As a quintessential class of smart materials, light-responsive materials offer the distinct advantage of precise control over their property changes and functions through noncontact light stimuli, facilitating unparalleled operational accuracy and remote programmable operation [1,2]. By converting light energy into mechanical work, functional changes, and information signals, photoresponsive materials establish their critical importance in advanced applications including photodeformable actuators [3], self-regulating optical systems [4], digitally programmed material platforms [5], and reconfigurable encryption devices [6].

As an important type of photoresponsive materials, photochromism refers to the reversible color change in response to light stimuli [7,8]. Photochromism compounds represent a significant subclass of chromogenic compounds, specifically referring to the reversible color-changing behavior triggered by optical radiation, whereas chromogenic compounds broadly encompasses color changes induced by various stimuli [9]. Owing to their outstanding reversibility, controllability, and response kinetics in the photoresponse process [10,11], photochromic materials have become indispensable for various high-end applications, spanning advanced photonic devices, optical sensing, and high-level information security [12–18]. As early as 1867, Fritsche discovered that yellow tetrabenzene could change color when exposed to light. However, it was not until the 1950s that the phenomenon, characterized by light-induced color variation, was formally defined as “photochromism”, following growing scientific interest. The earliest photochromic compounds were identified by Hirshberg during his studies on spiropyran (SP) derivatives, who became recognized as the pioneers of photochromism [19–21]. In the 1960s, military and commercial interests prompted scientists to conduct further research, leading to the development of the conceptual and theoretical foundations in photochromism field. Numerous new photochromic molecules were also discovered, laying the groundwork for future advancements.

Photochromism materials are classified into inorganic and organic systems according to their chemical composition. Inorganic photochromic materials are renowned for their exceptional fatigue resistance and environmental stability, with properties such as oxidation resistance, tunable coloration, and controllable fading kinetics making them valuable for specific applications [22–24]. However, the current commercial landscape is predominantly dominated by organic photochromic materials. A diverse range of organic molecular systems, including azobenzene, SP, fulgide, and diarylethene, have been extensively developed [25–28]. Their key advantage lies in highly tailorable molecular structures and superior processability, which not only makes them the material of choice for widespread commercial products such as photochromic lenses but also enables their use in flexible electronics and biomedicine [29–31].

With the wide application of organic photochromic materials, it has been found that a single light-responsive material is insufficient to meet the demands of multiple practical applications. Especially in harsh environments, it is difficult for single light energy to precisely control the behavior or reaction rate. Taking these factors into account, scientists began to consider whether other stimulus conditions could be utilized to further enhance the performance and intelligence of materials. This marks the original development of gated photochromism, in which the term “gate” refers to the use of external conditions to influence or control a photochromic process, thereby enabling its triggering or modulation by stimuli beyond light alone. For example, as early as the 1970s, Wilson and Drickamer [32] demonstrated the pressure-induced chromism of SP, revealing the fundamental coupling between mechanical force and photochromism. In the 1980s Fujishima and his collaborators [33,34] conducted pioneering work on photo-/electrochemical dual-stimulus-responsive systems. These early contributions served to lay a crucial foundation for the multimodal regulation of photochromic behaviors, thereby forming the historical roots of the field development [35–37]. In recent years, the development of multigated photochromic materials has been paving the way for transformative applications. The multigated mechanism provides superior controllability over conventional photochromism, holding great promise for revolutionizing advanced fields such as chemical artificial intelligence and biomimetic materials [38–41]. This potential is exemplified by their unique value in chemical AI, where they enable both Boolean and fuzzy logic processing and support the development of neuromorphic engineering in wetware through optical signal modulation.

**Fig. 1.**
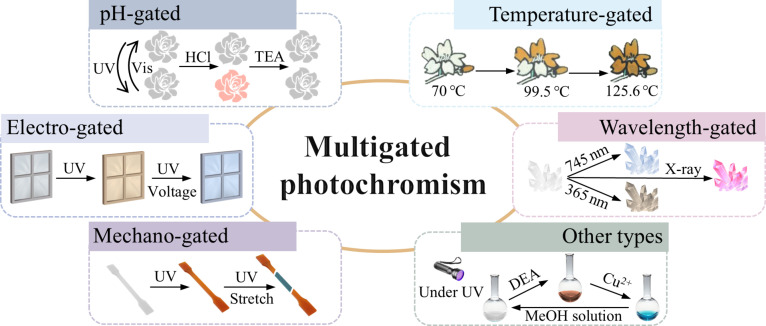
Schematic diagram of multigated color switching.

Briefly, organic multi-gated photochromic materials offer precisely controllable and highly programmable properties, which underpin their immense potential for future advanced applications. The ease of functional group modification and the tunability of the molecular structure endow the material with the ability to respond to additional stimuli. This capability not only improves its performance but also holds great promise for a broad spectrum of applications, such as flexible devices, sensors, actuators, and information storage [42–44]. This article mainly focuses on organic multigame photochromic materials (with gating modalities such as proton, voltage, mechanical force, temperature, and wavelength), which centers the discussion around their systematic design strategies and elucidates the substantial application potential of such precise regulatory properties in cutting-edge fields such as chemical sensing and dynamic information display. Figure 1 serves as a graphical abstract, concisely capturing the relationship between different gating mechanisms and their functional applications, thereby enhancing the reader grasp of the material versatile capabilities. In addition, some rare multiresponse photochromic materials are supplemented. Finally, the probable future development trends of these materials are prospected. All the molecules mentioned in the article are systematically summarized in Table 1. We believe that this review will contribute to both the fundamental understanding and practical advancement of gated photochromic systems, promoting their broader application across diverse scientific and technological fields.

**Table 1.** The table summarizes some basic properties and gated methods of the compounds appearing in the paper and marks the corresponding serial numbers at the abbreviations of the corresponding compounds

Number	Compounds	Excitation wavelength	Recovery wavelength	Original maximum absorption	Gating type	Forms	New maximum absorption	Color change	Ref.
1	HSp-PEGMA	UV	–	–	H^+^	Solution	ca. 440 nm	Yellow → colorless	[54]
2	SP−TPE−SP	365 nm	Vis	604 nm	HCl	Solid	–	Light yellow → luminous yellow	[55]
3	SP-PSF	365 nm	Vis	–	HCl	Solid	–	Gray → orange	[56]
4	SP-7	365 nm	Dark	540 nm	TFA	Solution	490 nm	Yellow → orange	[57]
5	DL-CHO	297 nm	*λ* > 500 nm	536 nm	TFA	Solution	636 nm	Colorless → green	[60]
6	o-ATE	365 nm	/	600 nm	TFA	Solid	545 nm	Turn colorless	[61]
7	TrPEF_2_-IPA	*λ* < 365 nm	Vis	462 nm	HCl	Solution	–	Colorless → red	[62]
8	Zr-MOP-1	365 nm	Dark	750 nm	−0.5–0.5 V	@PAN	621 nm	Brown → luminous green	[69]
9	SETV	365 nm	Dark	402 nm	−1.08–0.88 V	Device	543 nm	Light yellow →brown	[71]
10	D/L-Cd-MOF	UV	–	609 nm	−0.95–1 V	MOF	372 nm	White → black	[72]
11	1o-DLE	365 nm	*λ* > 490 nm	–	880 mV	Solution	–	Blue → green	[74]
12	3Tp-2o	UV	–	–	0.75 V	Solution	615 nm	Colorless → blue	[78]
13	Tz-1a	–	436 nm	–	0.32 V	Solution	–	Yellow → blue	[79]
14	Ru-1o	365 nm	650 nm	679 nm	−0.2–0.6 V	Solution	1,272 nm	–	[81]
15	ap-Fc	365 nm	–	–	Oxidized	MOF	ca. 540 nm	Colorless → red	[82]
16	NDI	365 nm	–	460 nm	−1.5–1 V	Solution	–	Light yellow → black	[83]
17	PAPDI-Br	365 nm	Vis	640 nm	−0.4–0 V	Solution	650 nm	Yellow → green	[85]
18	PEDOT:PSS	–	–	–	Voltage	Solid	–	Light blue → dark blue	[87]
19	SPDN	365 nm	Vis	580 nm	Stretch	Polymer	–	Light yellow → blue/purple	[91]
20	SPBCl	UV	Vis	584 nm	Grinding	Solid	–	Yellow green → green	[92]
21	Ph3SP	UV	Vis	580 nm	Grinding	Powder	570 nm	Red → dark red	[93]
22	NP5	UV	Vis	–	Stretching	Film	–	Colorless → yellow	[94]
26	NDP	UV	–	ca. 650 nm	Ultrasound	PMA-1	ca. 550 nm	Turn blue	[95]
27	SPO2	365 nm	–	615 nm	Grinding	Solid	605 nm	Light yellow → blue	[96]
28	CR-OB-CR	254 nm	Vis	550 nm	Ultrasound	Solution	564 nm	Colorless → dark red	[98]
29	DAEPI-o	365 nm	500 nm	–	Grinding	Solid	ca. 580 nm	Light yellow → yellow	[99]
30	DAESNH	460 nm	650 nm	–	20–70 °C	Coassembly	ca. 480 nm	Yellow → orange	[107]
31	XG	365 nm	–	–	80 °C	Powder	570 nm	Yellow → dark purple	[108]
32	ZnipbpBr	365 nm	*λ* > 400 nm	–	90 °C	MOF	652 nm	Yellow → Light green	[109]
33	TrPEoPO	UV	Vis	–	95 °C	Amorphous	460 nm	Colorless → yellow	[110]
34	FTrPE-ol	365 nm	Vis	–	70 °C	Aggregation	466 nm	Colorless → yellow	[111]
35	Si–O–Si	UV	–	–	31–45 °C	PDMS	–	Red or colorless → blue	[112]
36	JU99	–	–	–	X-ray	Solid	620 nm	Colorless → blue	[117]
37	TmTPC-1	UV	–	ca. 470 nm	X-ray	Solid	–	Colorless → dark green	[118]
38	Cdm-BTC	365 nm	–	ca. 600 nm	X-ray	Solid	–	Colorless → light blue	[119]
39	1a/2a-R	405 nm	–	–	Vis	Crystal	–	Colorless → orange	[120]
40	SO_2_-1a	405 nm	560 nm	554 nm	Vis	Solution	–	–	[121]
41	DTE-1	450 nm	600 nm	ca. 600 nm	Vis	Solution	–	Pale yellow → blue	[122]
42	AD(3)	530–560 nm	460 nm	–	Vis	Solution	–	Orange → yellow	[123]
43	BF_2_-1	570 nm	450 nm	–	Vis	Solution	–	Pink → orange	[124]
44	NN-Z	625 nm	450 nm	–	Vis	Solution	–	Purple → colorless	[125]
45	D1	520 nm	–	556 nm	Vis	Film	–	Dark purple → colorless	[128]
46	DE-g	808 nm	365 nm	580 nm	NIR	Integrated substrates	–	Purple → colorless	[129]
47	PP-5MR	785 nm	–	615 nm	NIR	Solution	–	Blue → yellow	[130]
48	M-1o	313 nm	480 nm	500 nm	Cu^2+^	Solution	–	–	[133]
49	PBC	254 nm	Vis	550 nm	Cu^2+^ or Zn^2+^	Solution	–	Pink → colorless	[134]
50	1O-B-Mes	365 nm	–	420 and 655 nm	F^−^	Solution	490 nm	Blue → yellow	[135]
51	1L-1o	297 nm	Vis	525 nm	CN^−^	Solution	ca. 420 nm	Purple → yellow	[136]
52	Zn-DPMNI-1	–	Dark	485 nm	DMF	Solid	–	Yellow → brown	[139]
53	Eu(ipbp)_2_·1	365 nm	–	669 nm	H_2_O	MOF	–	Yellow → light green	[140]
54	SP-AAO	UV	Vis	550 nm	SO_2_	Solid	–	Pink → light yellow	[141]
55	SP-DBU	UV	Vis	–	CO_2_	Solution	ca. 400 nm	Yellow → red	[142]
56	Cd-MOF-1	365 nm	Dark	476 nm	DEA	MOF	–	Light yellow → purple red	[143]
57	Red-CF(1)	470 nm	–	450 nm	Intensity	Solution	480 nm/750 nm	Orange → yellow/green	[146]
58	DASA-PCL	Vis	Dark	595 nm	Intensity	Film	–	Dark → colorless	[147]
59	ZnRho	365 nm	Vis	ca. 550 nm	Zn	Solution	ca. 550 nm	Light yellow → light red	[148]
60	PiPO-TTv	UV	–	–	0–1.5 V	Device	–	Colorless → purple → blue	[150]

DMF, *N*,*N*′-dimethylformamide.

## Design Strategies and Classification Mechanisms

The design strategy for multigated photochromic materials primarily focuses on the effective integration of photochromic units with other stimulus-responsive modules to achieve precise control over the photoisomerization process [45–47]. In contrast to traditional single systems, multigated photochromic materials also incorporate diverse responsive units at the molecular or supramolecular level, thereby constructing intelligent material platforms with multi-input and output logic capabilities. These design strategies fall into two main categories: (a) intramolecular functionalization, modifying the core photochromic structure with stimulus-responsive groups, and (b) intermolecular assembly/compositing, embedding the molecules into platforms such as polymers, metal–organic frameworks (MOFs), or supramolecular systems to leverage their unique structures and interactions for multiresponse behavior.

Building upon these design strategies, a clear classification framework emerges. Multigated photochromic materials are systematically delineated according to the nature of the applied external stimulus, mainly including pH-, electro-, mechano-, and temperature-gated systems [48–51]. Each gating class is characterized by a unique pathway for structural alteration, such as proton-induced (de)protonation, voltage-driven redox reactions, mechanoresponsive bond scission or phase transformation, and thermally regulated aggregation-state changes. These mechanisms furnish essential switching capabilities for the photoisomerization process at the molecular scale, thereby establishing a foundation for the practical utilization of these materials in sophisticated application environments. The incorporation of multiple stimulus responsiveness, in particular, substantially expands their potential application domains.

**Fig. 2.**
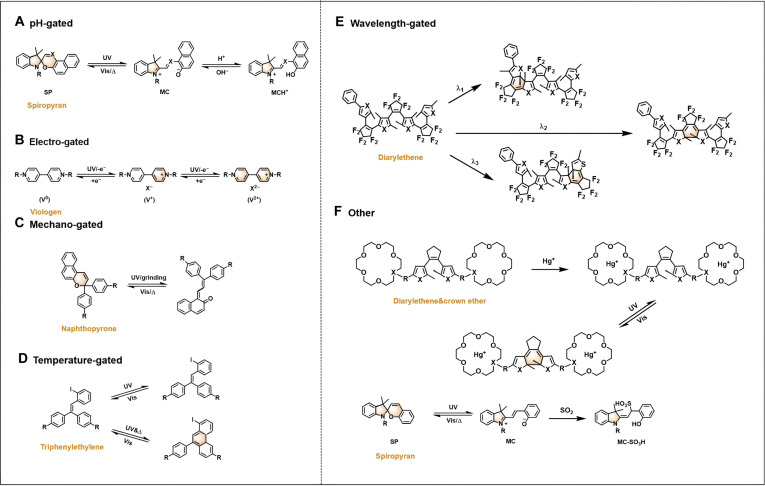
Classification of some typical multigated photochromic systems. (A) The isomerization of SP under UV–Vis light irradiation and different pH environment. (B) Photochromism of viologen driven by electrons. (C) Isomerization of naphthopyrone either under UV or grinding. (D) Photochromism of triphenylethylene occurred under UV irradiation and heat. (E) Isomerization of diarylethene under irradiation with 3 different wavelengths. (F) The photochromism of diarylethene with crown ether and SP activated by the induction of Hg^+^ and SO_2_ vapor, respectively

Owing to the distinct mechanisms across different systems, we have meticulously summarized them (Fig. 2). This illustration delineates the characteristic molecular transformation pathways for representative examples within each category, providing readers with an intuitive and clear visual guide to better comprehend the underlying mechanisms discussed throughout this review. Distinguishing itself by a systematic consolidation of design strategies and operational mechanisms, this review underscores the application prospects of multigated photochromic materials across multiple frontiers, pointing out key future research directions and potential breakthroughs for researchers.

## pH-Gated Photochromic Materials

pH-gated photochromic materials have garnered considerable attention due to their reversible color and structural changes modulated by protons. These materials not only retain the intrinsic photoresponsive characteristics of conventional photochromic systems but also incorporate pH as an additional control dimension, enabling more precise regulation of photochemical processes in complex environments. The fundamental mechanism involves the introduction of protons (H^+^), which can induce structural isomerization of the photochromic unit. For instance, protonation processes alter the molecular conjugation system, thereby modulating its light absorption properties and color. Currently, the most extensively studied proton-gated photochromic system is the SP and diarylethene system.

The pH-gated platform based on SP stands as a classic and one of the most extensively studied prototypes [52]. The core mechanism depends on the photo promoted cleavage of its C–O bond, initiating a reversible isomerization via ring-opening from the colorless, closed-form SP to the colored, open-form merocyanine (MC). When photochemical process encounters protons (H^+^), it is complicated into a richer response: The binding of H^+^ to the phenolate site of the MC form generates protonated MC (MCH^+^), which, in turn, causes a marked spectral shift and vivid color change [53]. Although this fundamental mechanism was elucidated decades ago, the research focus has since shifted from fundamental observation to the strategic molecular engineering and material design of SP. Therefore, this section centers on highlighting recent, representative design strategies and novel applications for pH-gated SP, offering insights into the evolving landscape of this field.

In 2018, Feeney and Thomas [54] prepared a hydrophilic polymer containing SP, which was combined with 3 methacrylates and hydrophilic monomers with different forms of charge (HSp-PEGMA). By rationally integrating SP with polymers, this work reveals the decisive influence of the microenvironment created by hydrophilic comonomers on the apparent p*K*_a_ (where *K*_a_ is the acid dissociation constant) of protonated MCH^+^. This discovery not only extends the operational window for efficient negative photochromism into the physiologically relevant pH range but also allows the regulating of material photoacid strength and response kinetics through simple adjustment of copolymer composition. It provides a key design paradigm and material platform for constructing new intelligent photocontrolled proton switches capable of stable operation in complex aqueous environments.

**Fig. 3.**
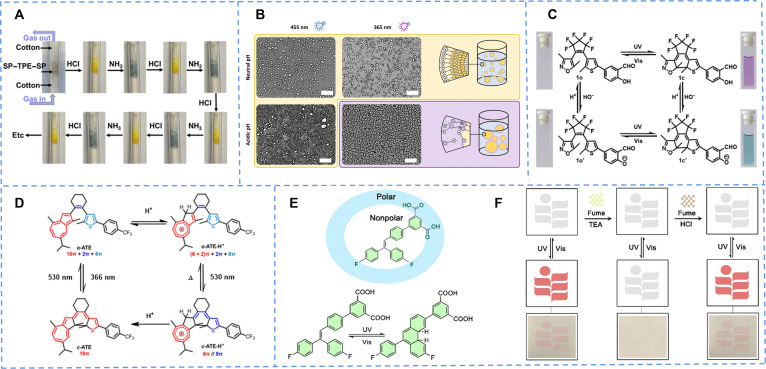
(A) The acidichromism in 5 cycles of solid-state SP–TPE–SP. Reproduced with permission [55]. Copyright 2018, American Chemical Society. (B) Light-controlled emulsion stability under different pH conditions: At neutral pH, irradiation with either UV (365 nm) or blue light (455 nm) leaves the emulsion structure unchanged. Under acidic conditions, however, UV light triggers demulsification, demonstrating a dual pH- and light-responsive smart switching behavior. Scale bars, 100 μm (transmission light micrographs). Reproduced with permission [57]. Copyright 2022, Wiley-VCH GmbH. (C) The isomerization and color changes of diarylethene 1o, 1o′, 1c, and 1c′ under UV irradiation or acid. Reproduced with permission [60]. Copyright 2014, Elsevier B.V. (D) The molecular structures and time-dependent UV–Vis absorption spectra of o-ATE, o-ATE-H^+^, and c-ATE-H^+^, which is caused by the addition of trifluoroacetic and TEA. Reproduced with permission [61]. Copyright 2020, Wiley-VCH GmbH. (E) Molecular structure of TrPEF_2_-IPA and the photoisomerization of TrPEF_2_-IPA. (F) Display of advanced anticounterfeiting properties by the photochromic paper in the presence of TEA and HCl. Reproduced with permission [62]. Copyright 2023, Royal Society of Chemistry.

Yin et al. [55] formed a new photochromic compound SP−TPE−SP by introducing SP into tetraphenylethylene (TPE). The solution changed from colorless to dark blue following ultraviolet (UV) irradiation. In contrast, the color change in the powdered solid was a stepwise progression from light yellow to green, ultimately reaching dark blue. When hydrogen chloride gas was introduced, the expanded free volume inside the polymer made small molecules be transported, and SP changed to the MCH^+^ structure. Then, ammonia was introduced to neutralize the acidity of the system, leading to the reverse color change. The construction of ample free volume not only enables efficient and reversible photoisomerization but also confers rapid and reversible acidichromic responses to gas HCl/NH_3_, offering a novel design strategy for developing next-generation solid-state smart materials—particularly in the fields of environmental sensing and anticounterfeiting encryption (Fig. 3A).

Distinct from conventional SP systems primarily studied in solution states, Sheng et al. [56] focused on the advanced design of solid-state and porous framework systems. Through sophisticated molecular engineering and innovative material composite strategies, they successfully anchored the proton-responsive behavior of SP within polymer matrices and highly porous frameworks (SP-PSF). This approach not only enabled highly sensitive detection of acidic gases in the solid state but also leveraged the reversible acidichromic characteristics to develop smart material platforms with practical application potential, such as intelligent acidic gas adsorbents and gas sensors.

Reifarth and colleagues [57] reported a dual pH- and light-responsive surfactant based on SP (SP-7). The innovation lies in incorporating the SP unit into the hydrophobic tail, rather than the traditional headgroup position, thereby enabling pH-gated photochromic behavior. The molecule reversibly switches between the hydrophobic SP form and the hydrophilic, protonated MCH^+^ form under acidic conditions, while its photoresponsiveness is substantially suppressed at neutral or alkaline pH. The color-changing mechanism involves the light-induced open ring of SP to generate MC, which is subsequently protonated to MCH^+^ in an acidic environment. This process not only alters the molecular charge state and solubility but also profoundly affects its surface activity and critical micelle concentration. A unique aspect of this design is the achievement of remote, dual control over emulsion stability: Demulsification is triggered only when acidic conditions and UV light irradiation are applied simultaneously, exhibiting behavior analogous to a molecular “AND” logic gate (Fig. 3B). Such intelligent surfactants hold broad application potential in controlled drug release, microreactor regulation, and smart colloidal materials.

The diarylethene system can also function as a proton-gated photochromic system. In contrast to SPs, which rely on intrinsic protonation for pH-gated photochromism, diarylethene systems typically achieve pH-gated behavior by introducing functional groups into their side chains or core structures [58,59]. This molecular design enables proton-induced modulation of electronic configuration changes, thereby regulating the photoisomerization pathway and absorption characteristics for precise control over color switching.

For instance, the hydroxyl protons in *o*-hydroxybenzaldehyde could be detached under alkaline conditions and recovered under acidic conditions. On the basis of this characteristic, the introduction of group on the side of diarylethylene could achieve acidichromism in photochromic transformation (DL-CHO) [60]. With the addition of triethylamine (TEA), the originally purple solution turned green, and the color returned to purple after the addition of trifluoroacetic acid (TFA) (Fig. 3C). It was found that the addition of alkali reduced the bandgap of diarylidene, resulting in the red shift of the maximum absorption wavelength by calculating the orbital energy. Its negative photochromic and proton-gated characteristics make it particularly suitable for cutting-edge applications such as high-security optical storage, intelligent sensing, and light-controlled molecular machines, representing a fundamental research breakthrough with significant application prospects.

Partial structures of azocyclenes may be affected by protons, so merging the azo moiety into photoswitch molecules leads to an unprecedented proton reaction. Azulene was incorporated into the backbone of diarylethylene to form o-ATE, exhibiting a novel proton-gated negative photochromic ring closure (Fig. 3D) [61]. It had a weak photochromic ability, but after adding TFA, its UV absorption at 300 nm was weakened and blue-shifted, and a strong absorption band from 350 to 550 nm appeared. Compared with traditional proton gating, the color change was achieved by controlling the concentration of H^+^ in the system, and the presence of H^+^ could also promote the process of photochromic process.

Briefly, SP and diarylethene represent 2 distinct strategies. SP inherently possesses acid-responsive sites, enabling fast and direct pH-regulated photoisomerization without the need for chemical modification. In contrast, diarylethene inherently lacks acid responsiveness and usually requires the introduction of responsive groups via chemical modification to achieve pH-gated functionality. This modification endows diarylethene with excellent thermal stability and fatigue resistance. Consequently, SP systems are more suitable for real-time sensing and detection applications where response speed is critical, while diarylethene systems demonstrate greater potential in optical storage and switching devices that demand long-term reversible operation and high stability.

The similar strategy has been extended to some other photochromic systems. Yu’s group [62] had designed a series of triarylethylene structures with stable photochromic properties, which could provide pH responsivity through structure modification (TrPEF_2_-IPA). Furthermore, they developed a novel porous photochromic crystal that demonstrates excellent fatigue resistance and cycling stability, showing great potential for practical applications (Fig. 3E). For the existence of carboxyl, the photochromism was responsive to TEA and acid. The photochromism could be quenched by TEA and recovered with the addition of dilute hydrochloric acid. With such stable and cyclable responsivity, applications in information encryption and multilevel anticounterfeit could be achieved. As shown in Fig. 3F, a new type of photoresponsive paper was prepared, on which the written information could be hidden by TEA fumigation and reappeared through photochromism when TEA was replaced by dilute hydrochloric acid.

In addition, spirooxazine compounds can undergo ring-opening protonation and develop color under acidic conditions, and their protonated products can reversibly revert to the closed-ring state upon visible light irradiation, forming a distinct acid/light dual-control response pathway [63]. On the other hand, as a classic photochromic unit, azobenzene also exhibits modulation of its photoisomerization process by protons: Studies have found that trace amounts of acid can substantially catalyze the cis-to-trans isomerization during its thermal relaxation [64]. The catalytic effect is attributed to the acid-promoted reduction of the thermal isomerization energy barrier, which effectively introduces a nonoptical “chemical gating” mechanism. These examples demonstrate that proton-gating strategies can be realized through diverse mechanisms, providing richer molecular design concepts for constructing intelligent multiresponsive material systems.

The entry of protons promotes the transformation of molecular structure, which is reflected color change in the macroscopical. Beyond their applications in traditional fields such as environmental monitoring and paper information encryption, these materials also demonstrate substantial potential in cutting-edge areas including visual detection, biosensing, and live-cell imaging. For instance, in microfluidic chips or cellular microenvironments, such materials can provide real-time, in situ optical responses to pH variations, thereby enhancing detection accuracy and response speed. These developments are expected to further drive the innovation of high-spatial-resolution visual sensing technologies and devices.

## Electro-Gated Photochromic Materials

Electro-gated photochromic materials refer to photochromic systems in which an electrical input modulates the photochemical switching process, for example, by altering the redox states, shifting the reaction equilibrium, or changing the activation barrier for cyclization. This concept differs from conventional electrochromism, where color changes are driven solely by an applied potential. Mechanistically, electro-gated photochromism is most commonly realized through (a) redox-mediated gating that generates or quenches chromogenic radical ions [65,66] and (b) electrochemically assisted structural transformations in π-conjugated switches [67]. The most prevalent electro-gated photochromic molecules are viologen and diarylethene, which demonstrate substantial potential for applications in areas including smart displays, information storage, and molecular logic devices.

Viologen is an organic compound based on 4,4′-bipyridine with a biquaternary ammonium structure, acting as an electron acceptor [68]. Under applied voltage or light irradiation, it undergoes 2 reversible single-electron reduction steps, changing its valence state from neutral to a divalent compound. During this process, the neutral species is oxidized and loses electrons, resulting in a color change.

**Fig. 4.**
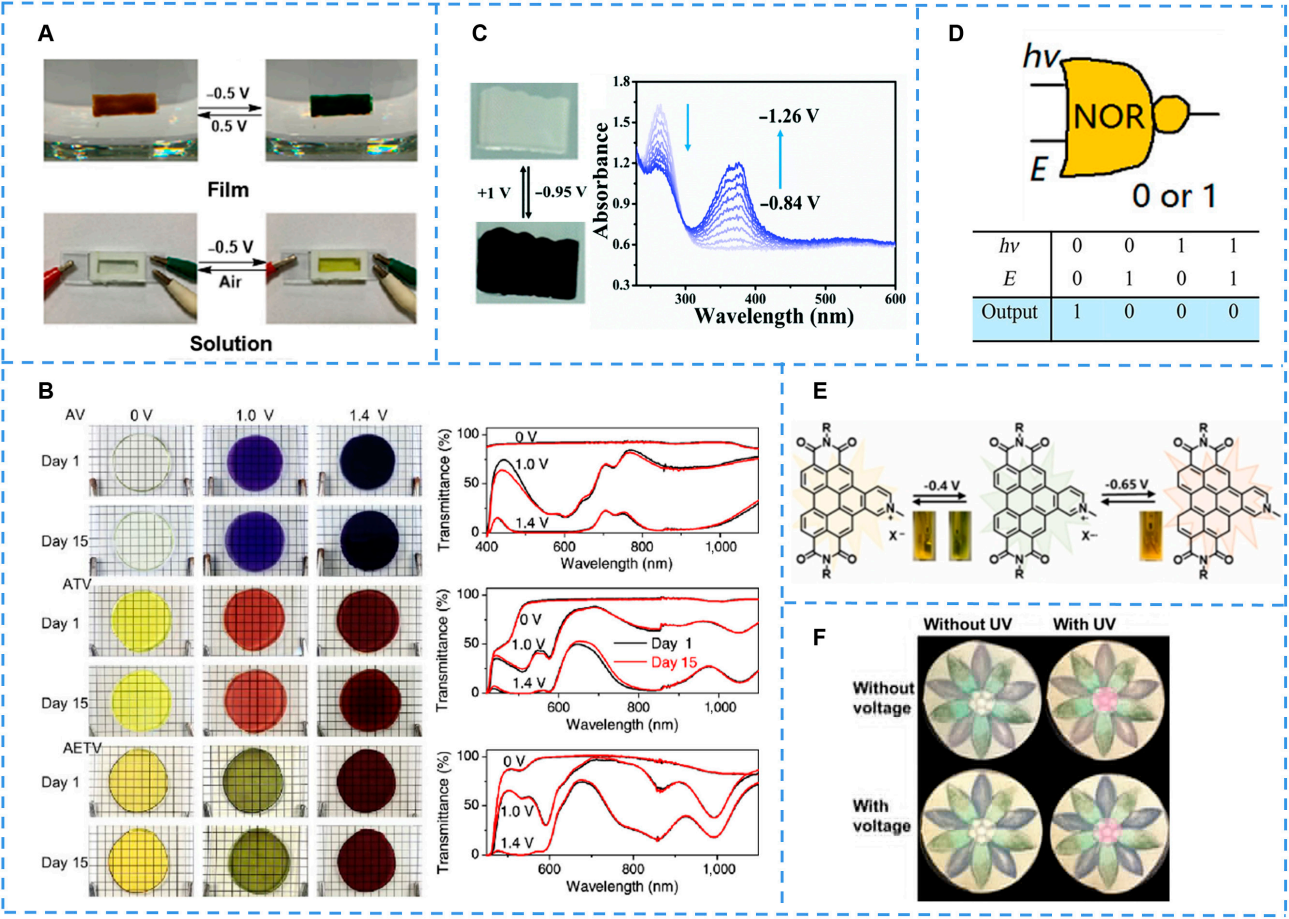
(A) The reversible electrochromic behavior of Zr-MOP-1 in a coated solid film and a methanol solution. Reproduced with permission [69]. Copyright 2022, American Chemical Society. (B) The obvious color change in Zr-MOP-1@PAN hybrid film under UV irradiation. Reproduced with permission [71]. Copyright 2022, American Chemical Society. (C) The reversible electrochromic behavior and the voltage-dependent absorption spectra of D-Cd-MOF in LiClO_4_ solutions. Reproduced with permission [72]. Copyright 2022, Royal Society of Chemistry. (D) The working principle of a multifunctional device with logic functions responding to photons and electrons as discrete inputs based on the electro-gated photochromic materials. The output signal is read through changes in absorption spectra, representing the logic states “0” or “1”, respectively. Reproduced with permission [81]. Copyright 2017, American Chemical Society. (E) The molecular species involved in the redox cycle. Corresponding color changes in solution taken in cuvettes are also shown in different time intervals. Reproduced with permission [85]. Copyright 2023, Elsevier Ltd. (F) Schematic diagram illustrating the working principle in a leather-based multistimulus-responsive chromic device: reversible and rapid color switching under light irradiation and applied voltage. Reproduced with permission [87]. Copyright 2021, Wiley-VCH GmbH.

By embedding viologen as a ligand into framework, Zang et al. [69] constructed a stable and sensitive MOF, which served as a basis for dual photo/electrical response materials. When doped into polyacrylonitrile, the resulting Zr-MOP-1@PAN mixed film changes color from light brown to light green under UV light. Moreover dissolved in solution of methanol, it could be used as a solvent-based battery: A remarkable change in color from transparent to green was observed (Fig. 4A). The application of an increasing potential to the Zr-MOP-1 solution resulted in enhanced absorption across the visible spectral range. Both the photochromic and electrochromic behaviors originate from the reduction of the viologen units to colored radical cations via electron transfer.

The introduction of conjugated moieties (such as thiophene) between the 2 pyridine rings of viologen derivatives can effectively extend the conjugation length, facilitating chromic behavior and enhancing functional performance [70]. The viologen molecule (SETV) originally did not have photochromic ability, which was later achieved by the incorporation of thiophene-derived bridges [71]. Hosted in a polyacrylamide hydrogel, these derivatives display both photochromism and electrochromism. The color changes in both cases originate from the formation of radical cationic species stabilized by the extended conjugation system. The electrochromic devices prepared by hydrogels not only had excellent chromism ability but also could exist stably in the air for a long time, and the transparency did not change basically (Fig. 4B). These multifunctional attributes make them highly attractive for practical applications in fields such as adaptive optics and energy-saving smart windows.

MOF materials have a periodic network structure, which promotes the transfer of electrons. The metal cadmium was used to construct a novel MOF material (D/L-Cd) featuring a 3-dimensional framework [72]. By ingeniously utilizing the electrochemical activity of viologen units, the material achieves reversible color switching from white to purplish-black under voltage stimulation. Its exceptional gating control characteristics make it an ideal platform for developing smart displays (Fig. 4C).

Diarylethylene also has the potential for electrochromism [73]. As early as 2003, Peters and Branda [74] connected thiophene rings at both ends of diarylethylene to achieve a color-changing material containing a double response (1o-DLE). At present, the most common view of diarylethylene systems is that cationic radicals are generated after the application of voltage, which causes the structural transformation [75]. There are also some people who believe that it is caused by the combination of the ring structure and free radicals [76,77]. However, regardless of the point of view, it can be attributed to a series of structural changes caused by redox reactions.

Irie et al. [78] introduced multiple thiophene rings to make diarylethylene easier to generate cationic radicals under the action of electric current (3Tp-2o). The voltammetry characteristic curves were tested for the compounds, and it was found that the closed-ring isomer produced a peak height in the plot, proving that the corresponding ring-opening isomer can be transformed into a closed-ring isomer by a redox reaction. By comparing the absorption spectra and the density functional theory (DFT) calculations, the result was obtained: Diarylethene derivatives undergo oxidative cyclization reactions when the radical cations of the closed-ring isomers are more stable than those of the open-ring isomers, and they undergo oxidative cycloreversion reactions when the radical cations of the open-ring isomers are more stable than those of the closed-ring isomers.

Hecht et al. [79] embedded heterocycles in the backbone of diarylethylene to enable an electrochemical reaction to close the ring under the stimulation of electric currents, thereby promoting a red shift in absorption (Tz-1a). While oxidative ring closure was facilitated by the presence of the terminal electron-donating substituent, photocyclization was inhibited because of an intramolecular charge transfer interaction. Interestingly, when inverting the configuration, it could only be performed in response to light stimuli and could not be achieved by electrochemical reactions, so these compounds should be of interest for designing multifunctional devices with logic functions responding to photons and electrons as discrete inputs.

Some diarylethylene systems linked by metal atoms have similar properties [80]. For example, the diarylethylene system was combined with metal Ru and fused ring compounds by coordination to achieve structural changes under the action of electric current (Ru-1o) [81]. Through this multivariate response method, a molecular logic gate was successfully prepared: When there was a current or light signal to stimulate the molecule, the corresponding spectrum was transformed into the number 1 after detecting the signal in the visible region, so that different signal outputs could be realized by changing the external environment (Fig. 4D).

Zhu et al. [82] reported a photochromic diarylethylene modified by ferrocene (Fc) with steric resistive ethylene bridges, which contained 3 functional parts: photochromic diarylethene unit, naphthalimide chromophore, and redox-active Fc (ap-Fc). In consideration of the large steric hindrance of Fc, the light color of the antiparallelotypal ap-Fc was blocked by chemical or electrochemical stimulation. If the iron in Fc was oxidized to trivalent, obvious discoloration occurred under the condition of UV light, and the quantum yield comparison was calculated to increase by nearly 50 times. When the oxidized structure was exposed to UV light, its solution changed from colorless to red, which was considered that the redox-gated process was realized.

It is a pity that the application of diarylethylene in electrochromism mainly stays at the molecular level, and there are not many practical scenarios. On the one hand, this may be due to the fact that the system often exhibits unidirectional changes (it can only change back to its original structure under light conditions). On the other hand, it may be due to the lack of a suitable substrate for application. Photochromic molecules exhibit excellent color-changing properties in polymers, potentially offering breakthrough pathways for future practical applications.

However, these 2 mainstream systems are not exhaustive. A variety of innovative systems have been attracting growing interest. Naphthalimide is one of the classic aromatic diimide compounds, which is widely used in multifunctional organic optoelectronic materials. The film that was formed by the self-assembly of naphthalimide was conducive to electron transfer and discoloration, and their performance can be enhanced with the support of a polymer matrix, thereby ensuring fast response and long-term stability in electrochromic devices (NDI) [83]. Under the stimulation of light and electricity, free radical anions were formed [84]. The originally transparent film became black when the number of anions reached a certain level, and the black color could be reversed by air or electrochemical redox substances.

Lin and colleagues [85] synthesized a series of pyridinium-annelated perylene diimide salts (PAPDI-X, X = I^−^, Br^−^, Cl^−^, NO_3_^−^, and PF_6_^−^). Through the introduction of charge-assisted anion–π interaction into aromatic imide derivatives, the stability of the generated free radical was enhanced (Fig. 4E). With the change in electron donating ability of anions, the system underwent the transformation from the interaction between nonchromogenic anions and π bonds to the charge transfer induced by chromogenic anions (PAPDI-Br). Among the anions mentioned above, the Br^−^ with moderate Lewis alkalinity could be involved in the electron transfer of PAPDI under the photo- or electrostimuli to form stable PAPDI-Br^−^ free radical anions and divalent anions, showing the unique double-response properties by photochromism and electrochromism based on the formation of free radical.

The electrochromic behavior of polythiophenes arises from an electric-field-driven redox reaction. Designing polythiophenes structure is an effective approach to achieve dual gated [86]. For instance, photochromic pigments and the electrochromic polymer were used to construct multiple stimulus-responsive units (poly[3,4-ethylenedioxythiophene] polystyrene sulfonate [PEDOT:PSS]) [87]. By integrating these functional colorants into distinct spatial regions of the same leather substrate, multistimulus-responsive chromism with independent operating characteristics was successfully achieved (Fig. 4F). This technology opens a practical new pathway for developing novel smart wearable devices with integrated capabilities for personalization.

In general, photochromic materials with electrical responsiveness can be divided into the following 2 categories: viologen and diarylethylene. The former has achieved many results in practical application, while the latter is mainly applied in molecular logic gates. Because the influence of electric current and light on the molecular configuration is obvious, the rate of discoloration can be completed in a few seconds, and even some molecular logic gates can complete the configuration transformation faster.

On the basis of their unique electro-/photo-dual-responsive characteristics, these materials offer reliable multistate color switching for intelligent displays because of their fast response and high cycling stability. This capability makes them well suited for applications in advanced display devices and dynamic information encryption. Moreover, these materials offer a promising platform for molecular logic gates. By allowing dual encoding of optical and electrical signals, they provide a tangible material foundation for building highly integrated chemical artificial intelligence systems. With the advancement of flexible electronics and wearable devices, these materials are expected to achieve further progress in these domain.

## Mechano-Gated Photochromic Materials

Mechano-gated photochromism describes a specialized process in which an external mechanical force serves as the “gate” to trigger or modulate a photochromic reaction. Unlike conventional photochromism driven solely by light, this dual-responsive mechanism involves the force-induced cleavage of specific labile bonds or the alteration of molecular packing, which subsequently enables the optical switching behavior. According to the design, these materials typically integrate photochromic units with mechanically sensitive molecular structures or embed them within force-transducing systems to achieve synchronous mechanical and optical responses. Specifically, the mechano-gated photochromism is primarily realized through 2 regulatory pathways: One involves direct modulation of the molecular structure, such as utilizing mechanical force to break unstable chemical bonds within the photochromic molecules [88]; the other involves indirect modulation of the microenvironment, for instance, by inducing phase transitions in molecular crystals or structural transformations in polymers through external force, thereby influencing photochromic behavior [89,90]. This chapter focuses on the molecular design, operational mechanisms, and emerging applications of mechano-gated photochromic systems, highlighting their potential in advanced fields such as stress imaging, multilevel anticounterfeiting, and rewritable optical storage.

**Fig. 5.**
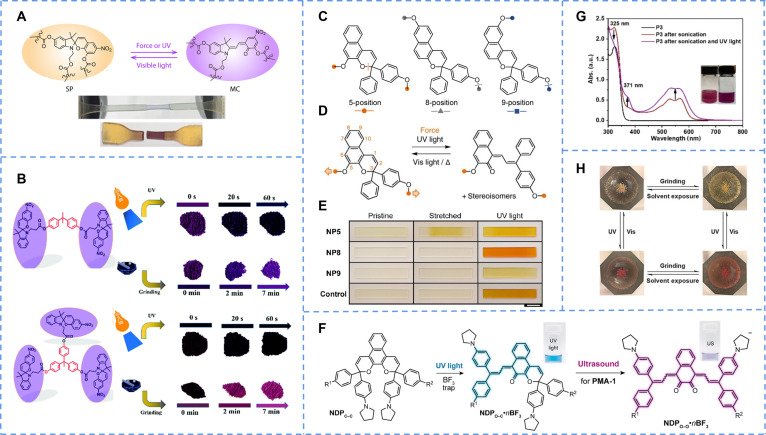
(A) The isomerization of SP and the corresponding color change in its doping polymers under the existence of external force. Reproduced with permission [91]. Copyright 2020, American Chemical Society. (B) The molecular structures and color changes of 2 different dendritic chromophores with applied UV light or grinding. Reproduced with permission [93]. Copyright 2021, Royal Society of Chemistry. (C) The molecular structures and (D) transformation of naphthopyran into a colored MC species under the existence of mechanical force. (E) Photographs of naphthopyran-incorporated PDMS film under the application of stretching or UV light. Reproduced with permission [94]. Copyright 2016, American Chemical Society. (F) Reaction scheme for the conversion of naphthodipyran to monomerocyanine NDP_O–C_·*n*BF_3_ in chain-centered polymer PMA-1 under UV light and ultrasound, together with corresponding color change. Reproduced with permission [95]. Copyright 2022, American Chemical Society. (G) UV–Vis spectra of naphthopyran under the application of ultrasonic or UV light. Abs., absorption; a.u., arbitrary units. Reproduced with permission [98]. Copyright 2023, Wiley-VCH GmbH. (H) The reversible photochromism and mechanochromism of benzo[b]phosphole alkynylgold(I) complexes. Reproduced with permission [99]. Copyright 2018, Wiley-VCH Verlag GmbH & Co. KGaA, Weinheim.

The traditional photochromic material SP can respond to physically stimuli. When the action of external force or light stimulation occurs, SP undergoes a open-ring reaction (SPDN) [91]. As illustrated in Fig. 5A, this process is initiated by cleavage of the labile spirocyclic C–O bond. The bond scission disrupts the connectivity of the molecule, resulting in an extended conjugated structure. In addition, the polymer structure is key to regulating its sensitivity, and the network structure markedly reduces the energy required to trigger the molecular change by optimizing force transmission and distribution.

Extensive research on dual-responsive SP derivatives has been conducted by Yang group. In 2020, photochromic derivatives with different steric hindrances were reported [92]. Introducing bulky groups reduced molecular packing density, providing sufficient free volume to facilitate efficient switching between the closed (SP) and open (MC) forms (SPBCl). The mechanochromism from light green to yellow upon grinding is primarily attributed to the incorporation of bulky groups, which increases intermolecular spacing and subsequently alters molecular packing modes and intermolecular interactions. This design strategy of modulating molecular structure through chemical modification to indirectly influence chromic behavior via external mechanical stimulation offers a promising pathway for developing advanced smart sensors and information storage materials.

The following year, the same group successfully synthesized dendritic SP molecules via esterification (Ph3SP) [93]. These dendritic photochromic chromogenic molecules had excellent photoswitching properties and gradually lightened the originally dark solid under the action of mechanical force (Fig. 5B). Different from typical strategies, the mechanochromism in this system stems from force-induced changes in physical aggregation and enhanced light scattering, rather than direct molecular isomerization.

Structurally analogous to SP, naphthopyran systems also exhibit mechanochromic behavior due to their unique spatial configuration. For example, Sottos et al. [94] had designed new naphthopyrans, which had 3 different forms of isomerism that reversibly transformed into colored MC species under UV light (Fig. 5C). These isomers differed in the location of the oxygen atoms, which resulted in different mechanochemical properties. DFT calculations revealed that compound NP5 could undergo bond cleavage and configuration change under minimal applied stress (Fig. 5D). Then, it was treated and added as a covalent doping to polydimethylsiloxane (PDMS). Stretching a PDMS film containing 5-bit linked NP5 caused the measured region of the material to turn orange–yellow, indicating mechanochemical conversion of naphthalene ether to cyanogen species (Fig. 5E). Tensile tests and DFT calculations showed that the mechanochemical activity of the 5-position substituted naphthalopyrane was stronger, so that the C−O pyrane bond and the external mechanical force were better aligned along the direction of the reaction coordinate, and then the color change was displayed on the macro level.

It is proved that the ring-opening only exists only at one side in naphthodipyran under the UV irradiation. To expand this concept, Robb and colleagues [95] demonstrated that naphthodipyran—which typically undergoes ring-opening on only one side under UV—could be endowed with dual-side ring-opening capability under mechanical force when covalently linked to polymer chains (NDP). Under the existence of ultrasonic, the photochromism was maintained, and the solution turned to purple. Compared with the pure UV irradiation condition, there was a red shift of the absorption peak, showing unique near-infrared (NIR) absorption properties (Fig. 5F). This groundbreaking study demonstrates the use of mechanical force to achieve a dual open-ring reaction, revealing a multimodal correlation between mechanical response and color change. This discovery not only expands the design strategy for mechanophores but also provides a new molecular foundation and pathway for developing intelligent polymers with multicolor response and stress visualization, such as stress-sensing coatings.

Spirooxazine represents another important class of mechano-gated photochromes. Gentili et al. [96] revealed that microcrystalline spirooxazine powder can undergo a color change from colorless to blue under external mechanical force (SPO2). This mechanical force promotes the cleavage of the C–O spiro bond and induces the molecular transformation to a planar MC configuration. The excellence strategy lies in the use of solid-state physical mixing and mechanical activation to efficiently integrate multiple stimulus-responsive properties into a simple solid system. This approach enriches the path for constructing solid intelligent materials and provides a highly inspirational prototype for developing novel sensors.

Rhodamine, a widely used fluorescent dye, has also been engineered into mechanochromic systems [97]. The rhodamine unit is conjugated to the photochromic dithienylethene unit, enabling the construction of a novel material CR-OB-CR through a relatively universal design strategy [98]. Under ultrasound conditions, the lateral groups of rhodamine were converted from ring-closed results to ring-open structures, while the colorless solution became a dark-red solution (Fig. 5G). A key feature of this molecule is the independent nature of mechano- and photoresponsive behaviors, and 2 types of stimuli can orthogonally and independently activate different molecular components, respectively. This design achieves dual gated material, providing a conceptual foundation for developing a new generation of multifunctional optical force sensors.

Photochromic benzo[b]phosphole alkynylgold(I) complexes represent another versatile dual-responsive system (DAEPI-o) [99]. Depending on the substituents they are connected to, different color variations could be produced. After grinding, with the existence of mechanical external force, the powder could give out a more obvious color change (Fig. 5H). Because of the donor–acceptor nature and the presence of aromatic and heteroaryl rings, they all had different interacted twist angles. Under the application of external force, the crystalline state of the molecule changed to an amorphous phase, and the change in the planar structure of the molecule was the cause of the mechanical discoloration behavior.

In summary, SP and naphthopyran derivatives currently represent the most prevalent molecular platforms for achieving mechano-gated photochromism [100,101]. For mechano-gated photochromic materials, distinctive molecular structure is prone to alterations in configuration or alignment under mechanical force and is highly easy to chemical modification. These properties collectively establish a solid foundation for advancing mechano-gated photochromic materials. Furthermore, it is evident that mechano-gated photochromism operates through 2 fundamental mechanistic strategies. Guided by these mechanisms, a growing number of materials possessing such characteristics are being designed and discovered, holding substantial potential for applications in high-density mechano-optical data storage and real-time stress-distribution mapping.

## Temperature-Gated Photochromic Materials

Temperature-gated photochromic materials represent a class of novel smart materials whose photochromic behavior is regulated by temperature variations. The key design challenge for such materials lies in balancing the intrinsic thermal stability required for the photochromic unit and the thermal responsiveness needed for temperature-gated functionality. As an optical switch, the photochromic state must remain thermally stable for reliable storage, whereas as a thermally responsive element, the same state needs to respond rapidly at a specific temperature to enable temperature-sensitive functionality. Briefly, temperature affects the reversibility and colorability of all those photochromic compounds, which results in mutual interference with photochromism during the forward excitation phase [102]. Two primary strategies are used to achieve temperature-gated photochromism: One approach involves the design and synthesis of molecules that integrate both photo- and thermoresponsiveness, while the other relies on indirect methods, such as modulating molecular alignment or leveraging polymer matrices [103–105]. These advanced photothermal materials hold significant promise for applications in smart windows, sensors, and anticounterfeiting technologies.

Of the early photothermal-responsive systems, one notable example involved the oxidation of tetraphenylpyrrole, through which Maeda et al. [106] obtained a dual-response dimer. The specific cause of color change was the dissociation of free radicals induced by light or heating, which, in turn, lead to structural alterations and thus imparted the ability to change the color. In recent years, advancements in photochromic systems have spurred the development of multigated materials. Among them, photochromic materials exhibiting thermal responsiveness have garnered substantial attention. By applying the aforementioned design strategies, scientists have successfully developed a series of systems possessing these integrated properties.

**Fig. 6.**
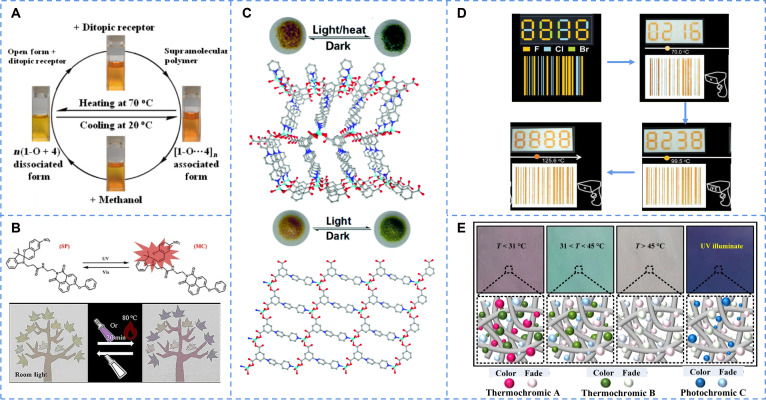
(A) The reversible photochromic and thermochromic behavior between different coassembly supramolecular polymers. Reproduced with permission [107]. Copyright 2019, American Chemical Society. (B) The isomerization of XG, causing the color change in paintings composed of grinded XG powders under UV light or heat. Reproduced with permission [108]. Copyright 2023, Elsevier B.V. (C) The 3-dimensional interpenetrating cationic frame and the 2-dimensional layered cation network of 2 types of Zn-MOFs, and the 3-dimensional frame exhibits an apparent color change under UV irradiation or heat. Reproduced with permission [109]. Copyright 2016, Royal Society of Chemistry. (D) Bar codes and digital encryption with different temperature under UV irradiation for multilevel information encryption. Reproduced with permission [111]. Copyright 2023, Elsevier B.V. (E) Paintings composed of thermochromic and photochromic powder under a variety of environmental temperatures. Reproduced with permission [112]. Copyright 2022, Springer Link.

Some temperature-gated photochromism molecules are derived from modifications of common photochromic molecules to introduce thermochromic ability. For example, a new series of photochromic barbiturates containing benzo[b]phosphorus pore oxides was successfully prepared, which had coassembly properties with dipore acceptors with bisdipyrimidine pyridine (DAESNH) [107]. The supramolecular assembly undergoes reversible switching between the open and closed forms upon light irradiation, accompanied by a shift in the absorption spectrum, leading to color changes. Furthermore, the coassembly system exhibits remarkable thermochromic behavior: The mixture [1O···4] changes color from orange back to yellow upon heating to 70 °C and reversibly recovers upon cooling (Fig. 6A). UV–visible (UV–Vis) absorption spectra reveal a decrease in the low-energy absorption band and an increase in the high-energy band during heating, indicating the disruption of hydrogen bonds and dissociation of the coassembled structure at elevated temperatures. This reversible thermochromic response originates from the temperature-dependent association and dissociation of the hydrogen-bonded supramolecular polymer, directly linking the macroscopic color change to the state of the coassembly. The strategy successfully achieves dual responsiveness and shows great potential for applications in smart-responsive materials.

Naphthalimide was added to SP to construct a novel SP-derivative XG [108]. The compound exhibits obviously photochromic and thermochromic phenomena in the solid state after grinding: Under UV irradiation or heating at 80 °C, the solid shows a color change from yellow to dark purple (Fig. 6B). The chromism mechanism originates from the isomerization of the SP unit from the closed-ring to the open-ring form. Grinding disrupts the tight intermolecular packing and hydrogen bonding, reduces crystallinity, and thereby enhances the thermal response performance, creating favorable conditions for isomerization in the solid state. On the basis of these high-contrast, reversible, and rapidly responsive thermochromic properties, this compound demonstrates broad application prospects in time-resolved information encryption and dynamic anticounterfeiting.

Coordination compounds represent a prominent approach for achieving multigated. Liang et al. [109] utilized the carboxylic acid bipyridine ligand (H_2_ipbpBr) to construct 2 novel Zn-MOFs. Research demonstrates that compound 1 (ZnipbpBr) exhibits temperature-gated photochromism, while compound 2 does not, a difference primarily attributed to their distinct structural configurations. The 3-dimensional interpenetrated framework of 1 facilitates a denser packing, resulting in a shorter distance between electron donors and acceptors. This structural feature promotes heat-induced electron transfer, leading to the formation of radicals and a visible color change. In contrast, the 2-dimensional layered structure of 2 hinders this process (Fig. 6C). These findings indicate that the donor–acceptor interactions in MOFs can be tailored through precise control over their topological structure and packing mode. Such temperature-gated materials show potential for applications in temperature sensing and smart windows.

The influence of temperature on a material extends beyond bond breaking to include changes in physical state, such as the transformation from crystalline to amorphous phases. Therefore, multigated is achieved with the help of morphological transformation, and triarylvinyl is not only a representative of photochromic materials but can also achieve this strategy. Yu et al. [110] designed a novel tristyrene derivative that could regulate the aggregation state by temperature (TrPEoPO). The tristyrene derivatives in the crystal state did not have photochromic properties but showed marked photochromic properties when heated to a molten state. A patterned quartz substrate, fabricated by filling its grooves with photochromic molecules in different states, enabled reversible pattern switching upon stimulation. Therefore, an interchangeable and rewritable optical information storage material could be realized by simply controlling the aggregation state of solids.

Building on the principle of the aggregation state regulation, Yu’s group [111] successfully introduced thermal gating into organic photochromic materials, constructing thermal activated photochromic materials (FTrPE-ol). These materials could change their aggregation states through temperature changes to realize the “on–off” control of the photochromism. When the material was heated, it was transformed from aggregation state to amorphous state. According to the crystal analysis and theoretical calculations, the phenomenon was caused by the breaking of lattice bond under heat, which led to the decrease in weak intermolecular interactions and easier photochromic cyclization reactions, finally manifesting more obvious photochromic phenomena. As shown in Fig. 6D, the valid information in the barcode prepared with that material could be only read under UV irradiation at a specific temperature. This deliberate design ensures that the information remains concealed and secured under all other ambient conditions, thereby enhancing its anticounterfeiting capability.

Wang et al. [112] constructed a composite coating system consisting of photochromic and thermochromic particles, vinyl-terminated PDMS and a siloxane skeleton (Si–O–Si). This coating uses an organic–inorganic doping strategy to achieve dual responsiveness to both external temperature and UV irradiation. The synergistic interaction of different photochromic and thermochromic pigments enables a 4-state color switching mechanism, whose principle lies in the reversible changes in the structure or electronic state of each pigment under external stimuli (Fig. 6E). PDMS-terminated vinyl not only reduced surface energy but also improved the durability of cross-linking reactions. The coating demonstrates broad application potential in the fields of tactile imaging and multicolor fabrics.

From the above summary, 2 primary design strategies have been established in the field of thermally gated photochromic materials. Although these strategies have facilitated applications in sensors and anticounterfeiting technologies, several critical challenges remain unresolved [113,114]. The narrow effective operating temperature range compromises performance stability under fluctuating thermal conditions. In addition, the relatively slow thermal response hinders their deployment in applications that demand rapid switching. These limitations—including constrained temperature thresholds, kinetic hysteresis, and insufficient cycling durability—collectively hinder the reliable deployment of such materials in practical applications such as high-precision sensors. Future research efforts will focus on the aforementioned strategies, aiming to broaden the operational temperature window and accelerate response kinetics, thereby facilitating their transition toward practical applications.

## Wavelength-Gated Photochromic Materials

Within the framework of multigated photochromic systems, optical wavelength serves as a fundamental control parameter that enables sophisticated material regulation. Through rational molecular engineering, these materials can be programmed to demonstrate distinct photoresponses upon irradiation at different wavelengths, exhibiting characteristic spectral shifts and color variations. This wavelength-dependent gated mechanism substantially expands the functional versatility of multigated systems. Such capability to achieve wavelength control opens promising ways for advanced technological applications, including high-security encryption platforms, reconfigurable photonic elements, and molecular logic gate [115,116]. This section systematically examines the molecular design strategies, unique multicolor switching behaviors, and emerging applications of wavelength-gated photochromic materials.

### X-rays

X-rays are electromagnetic waves characterized by higher energy and shorter wavelengths than UV light and are widely utilized in medical and industrial testing. X-rays, owing to their high photon energy, can induce bond cleavage and structural transformations in specific compounds. This provides the basis for realizing x-ray-gated photochromism, representing a distinct optical response phenomenon activated by high-energy radiation. This opens up new avenues for developing novel functional materials and pioneering frontier technologies.

**Fig. 7.**
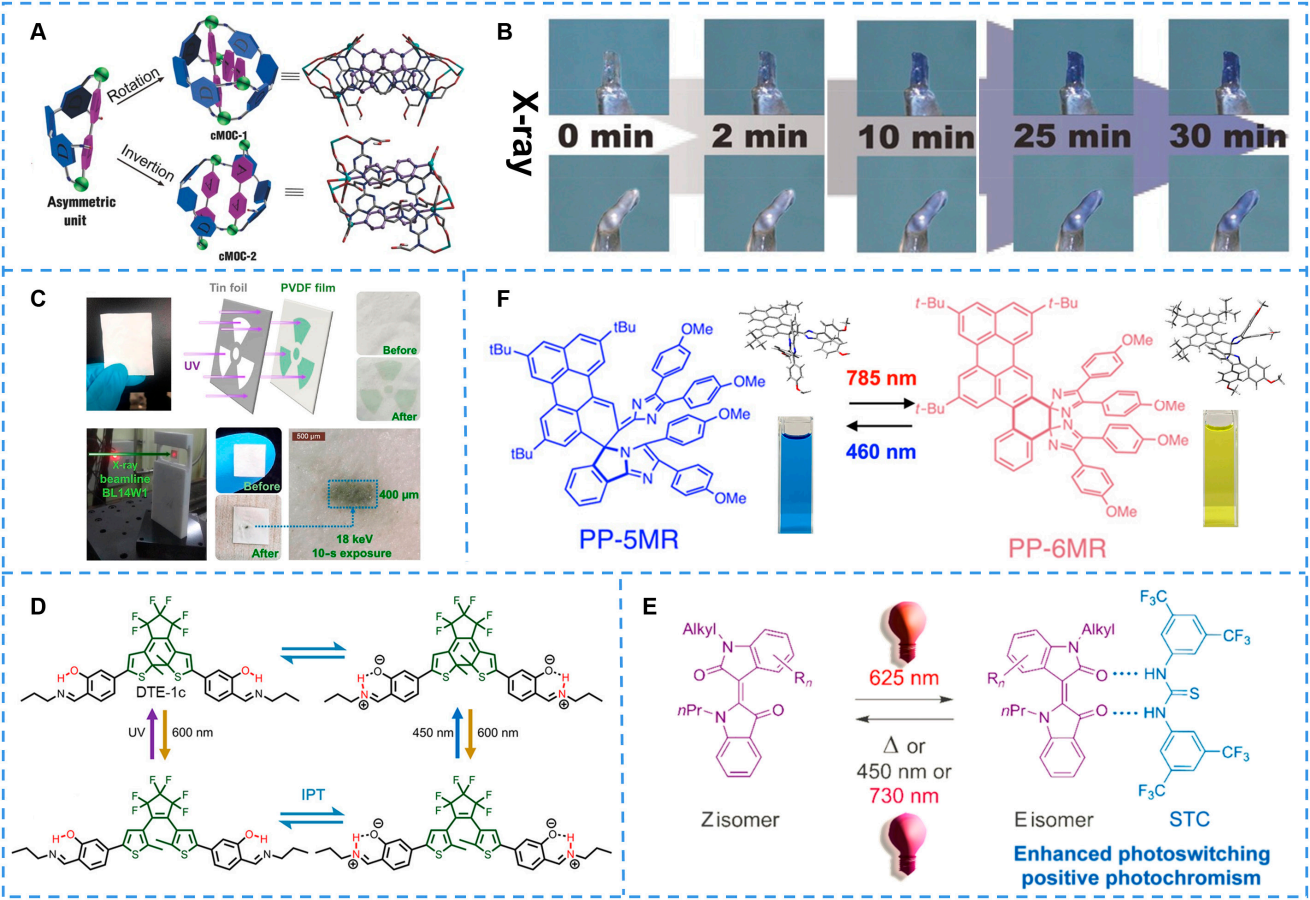
(A) The molecular structures and (B) the photochromism under x-ray irradiation of cMOC-1 and cMOC-2. Reproduced with permission [117]. Copyright 2015, Wiley-VCH Verlag GmbH & Co. KGaA, Weinheim. (C) The photograph and configuration of the UV made by TmTPC-1@PVDF membrane. Reproduced with permission [118]. Copyright 2021, American Chemical Society. (D) Reactions of the photochromic dithienylethene system conferred by the IPT process. Reproduced with permission [122]. Copyright 2019, American Chemical Society. (E) Visible-light (red)-responsive photoswitching of N-alkylated indirubin, enhanced through supramolecular complexation by Schreiner’s thiourea organocatalyst (STC). Reproduced with permission [125]. Copyright 2021, American Chemical Society. (F) The isomerization between BN-ImD and PhPe-ImD under visible and NIR light, together with the singe crystal analysis and color change(*t*-Bu:tert-Butyl, OMe:Methoxy). Reproduced with permission [130]. Copyright 2023, American Chemical Society

Metal–organic complexes are difficult to realize photochromism under room temperature X-rays, but Zhang and Wu [117] prepared 2 calcium-like metal–organic complexes (cMOC-1 and cMOC-2), which could change color in solid state under x-ray irradiation at room temperature (JU99). The substantial structural differences between 2 complexes stem from their different symmetry, manifested as the symmetrical structures in the former and crowded 2-dimensional structures in the latter (Fig. 7A). This structure allowed the former to change from light yellow to bluish violet in lower energy x-rays, but the latter required higher energy and longer excitation time to photochromism contrast to the former (Fig. 7B). The most remarkable advantage of the compound was that the structure of the compound did not change when irradiated by UV light and could only respond specifically to x-rays. The way to achieve x-ray-induced photochromism by imitating the host–guest system provides a strategy for novel materials.

Some molecules exhibit photochromic behavior under both x-ray and UV light due to their relatively low bond dissociation energy, presenting a practical alternative for achieving dual excitation. Lin et al. Prepared a carboxylic acid complex TmTPC-1 with reversible photochromic transition from colorless to green under both x-ray and UV light. Another compound, TmTPC-2, did not have color-changing ability [118]. By comparing their x-ray diffraction spectrums, it was found that the former had a more symmetrical structure in single crystals, which benefited electron transfer, promoting the transition of electron π and enough conductivity for the formation of free radicals. To realize further application, a kind of UV detection membrane and radiation detector were prepared by combining TmTPC-1 molecule with polyvinylidene difluoride (PVDF). As shown in the Fig. 7C, the visible color changes and corresponding patterns generated as x-rays pass through the film provide a more convenient observation method for x-ray imaging applications.

Furthermore, Liu et al. [119] synthesized 3 coordination polymers with metal ions and violet essence, all of which could respond to the dual irradiation of UV and x-ray to achieve photochromism (Cdm-BTC). Those compounds respectively changed from colorless to violet, blue, and light blue under UV light but all to light blue under x-rays. Their reversible photochromic properties under light irradiation could be attributed to the production of light-induced free radicals.

Nevertheless, the development of organic photochromic materials responsive to x-ray excitation remains limited because the inherently weak absorption of high-energy x-rays by organic molecular frameworks and the propensity of x-ray irradiation to cause irreversible molecular degradation. In contrast, inorganic systems demonstrate superior stability under x-ray exposure, offering valuable insights for innovative material design. Strategies such as constructing organic–inorganic hybrid structures could enable efficient x-ray photochromism, potentially opening new pathways for advanced optical applications.

### Visible light

Visible light excitation serves as the primary mechanism for achieving photochromic responses across multiple wavelengths in advanced material systems. The core of the visible light excitation system is how to excite photochromic molecules at a small energy to make them change their structure. Visible light excitation presents distinct advantages over UV radiation in biomedical and health-related applications owing to its superior biosafety profile. This inherent safety characteristic establishes visible light as the preferred stimulus for photoresponsive systems operating in biological environments. In addition, the availability of diverse modulation mechanisms further enables multifaceted approaches to information encryption, anticounterfeiting, and security applications. This section excerpts photochromic molecules under different wavelength excitations.

Currently, the most common photochromic systems excited by visible light are mainly concentrated in the diarylethylene system. Irie and colleagues [120] were among the first to introduce visible light into the photochromic system. Colorless crystals appeared orange under 405-nm irradiation, proving that the energy required for excitation could be reduced by rationally designing the molecule (1a/2a-R). In 2014, Irie and colleagues [121] demonstrated through numerous experiments that the easiest way to achieve photochromic excitation by visible light was to extend the π-conjugated length by attaching aromatic ring compounds with highly expanded π-electronic systems (SO_2_-1a). Zhu et al. [122] further extended the strategy proposed by their predecessors to achieve visible light excitation with the help of intramolecular interaction forces (DTE-1) (Fig. 7D). The new system not only worked in polar solvent systems but also showed its effectiveness in polymer gel systems.

Woolley et al. [123] substituted hydrogen on the methoxy *p*-benzene ring to obtain an amidezobenzene derivative [AD(3)], which led to an obvious red shift in the *n*–π* band of the trans isomer, thereby replacing the original UV-controlled discoloration reaction. The system could undergo structural changes under the irradiation of green light and recover under the irradiation of blue light, realizing the change between cis and trans isomers. It was rare to realize cis–trans isomerization of isomers by 2 specific wavelengths of light, and the isomers had thermal stability which made them good bidirectional optical switching materials. Another azobenzene complex was reported, which could be induced isomerization without harmful UV light (BF_2_-1) [124]. The compound changed from transformative form to a cis form under 570-nm irradiation, and the color changed from bright purple to light orange. Through analysis of the nuclear magnetic resonance spectroscopy, the benzene ring was far away from the BF_2_ group during isomerization, indicating that the phenomenon observed in the compound was indeed a trans/cis isomerization process.

Dube et al. [125] used alkyl groups instead of acidic NH protons to convert indigo into optical switches responding to red light (NN-Z). They designed and studied the photoswitch characteristics of different indigo derivatives. All derivatives showed obvious absorptions in solution, with maximum absorption peaks around 600 nm, which made them appear blue to the human eye. Under continuous irradiations of different wavelengths, no spectral change was observed in the unsubstituted indigo 1c at ambient temperature. In addition, indigosin derivatives were convertible to E-isomers at 625 nm. Moreover, the photochemical properties of indigo were substantial enhanced through supramolecular interactions after the addition of a hydrogen-bond acceptor (Fig. 7E).

Furthermore, some ingeniously structured photochromic systems can even be regulated by light of different wavelengths to control distinct states, thereby enabling multicolor-switchable optical behavior [126,127]. Such materials not only expand the dimension of visible light regulation but also offer new pathways for multicolor displays and dynamic information encryption. For instance, Sun et al. [128] utilized the selective linear-to-cyclic isomerization of donor–acceptor Stenhouse adducts (DASAs) upon irradiation with visible light of specific wavelengths achieving wavelength-discriminated photochromic responses by precisely matching the irradiation wavelength with the absorption bands of different DASAs (D1). By combining multiple DASAs with complementary absorption peaks and nonphotochromic organic dyes, the authors constructed a material system that exhibits uniform absorption in the dark state. Under monochromatic light excitation, selective bleaching creates an absorption gap, enabling adaptive color matching to the background.

In summary, visible-light-excited photochromic systems, achieved through sophisticated molecular design such as π-conjugation extension, donor–acceptor functionalization, or intramolecular proton transfer, successfully lower the excitation energy from the UV to the visible region. This shift not only enhances biosafety in biomedical applications but also significantly expands the precision and versatility of optical control. By enabling selective excitation at distinct wavelengths within the visible spectrum, these systems exhibit distinguishable multistate switching behaviors. This wavelength-dependent tunability endows visible-light-gated photochromic materials with unique and significant potential for applications in high-security information encryption and multicolor displays.

### NIR light

NIR light, characterized by its deep tissue penetration, low phototoxicity, and excellent biocompatibility, represents an ideal excitation source for photochromic materials. The common association of NIR with negative photochromism provides a unique optical switching behavior, opening new pathways for developing high-contrast optical devices and reversibly regulated systems within biological organisms.

For example, Kitamura et al. [129] coated an ultrathin polymer film doped with diarylethene derivative on a glass substrate with gold nanoparticles using a 2-photon ring open photochromic reaction of a diarylethylene derivative under NIR light (DE-g). This special plate could promote the ring-opening transition of diarylethylene under continuous NIR waves with bleaching effect to achieve negative photochromism.

Binaphthyl-bridged imidazole dimer (BN-ImD) were reported by Moriyama and Abe [130], which had 2 isomers: The colored and colorless isomers with a 5- and 6-membered ring were named 5MR and 6MR, respectively. BR was a special intermediate, which thermally isomerized to 5MR or 6MR by an intramolecular radical coupling reaction. Although the intermediate material could be converted into different isomers by different wavelengths of light, the controllable range between them was short and difficult to control. Therefore, on the basis of the original different wavelength manipulations, it was improved to achieve a larger wavelength control range between the 2 isomers. Modified PP-5MR was blue solution with a absorption peak at 615 nm, and the color of the solution changed from blue to yellow upon irradiation with a 250-mW 785-nm light (Fig. 7F). Higher photochromic efficiencies should be achieved through modifications of the molecular architectures and electronic structures of binary-bridged imidazole dimers.

The negative photochromism caused by the near infrared is not rare. Dihydropyrene was a negative photochromic molecule commonly used in the preparation of synthetic resins, vat dyes, and dispersion dyes. Hecht et al. [131,132] conducted a lot of researches on this system and designed a series of derivatives with negative photochromic phenomena based on different strong donor–acceptor chromophores. Under the NIR light, the molecular structure changed, leading to the color change in the solution. These molecules could be switched entirely with far-red or NIR light, and the process was affected by factors such as the intensity of the substituents, environmental polarity, and others.

Overall photochromic systems regulated by multiple wavelengths have been demonstrating considerable application potential due to their precise excitability across different spectral regions. In the future, light-controlled systems operating in distinct wavelength bands are expected to play critical roles in their respective fields of strength. With ongoing innovations in molecular engineering and material design, wavelength-gated photochromic materials are poised to enable integrated and intelligent applications across various cutting-edge domains—such as sensing, imaging, information storage, and biomedicine—thereby driving the advancement of next-generation photoresponsive material systems.

## Others

While the preceding sections have systematically detailed mainstream multigated photochromic materials, the scope of this field continues to expand with the emergence of numerous less common yet functionally novel gated systems. These systems serve as an important complement to the established ones. This section will focus on these unconventional gated photochromic materials. Owing to their unique triggering modes, they may play critical roles in specific application domains, such as highly selective chemical sensing and visualization of microenvironments, demonstrating distinctive potential. Exploring these unconventional systems will contribute to a more comprehensive understanding of the diversity and future development prospects of multigated photochromic materials.

### Ion-gated photochromism

Photochromic materials with ion-gated responsiveness have demonstrated significant potential in fields such as environmental monitoring and information encryption, owing to their high selectivity and responsiveness toward specific ions. These materials typically rely on the synergistic interaction between photochromic units and ions. Their color-changing behavior is not only triggered by light but can also be modulated through coordination or electron transfer processes involving ions and molecular structures, enabling multiple logic inputs and outputs. Ion-gated responses can generally be categorized into 2 types: metal ions and inorganic anions. This section will discuss both systems in detail.

**Fig. 8.**
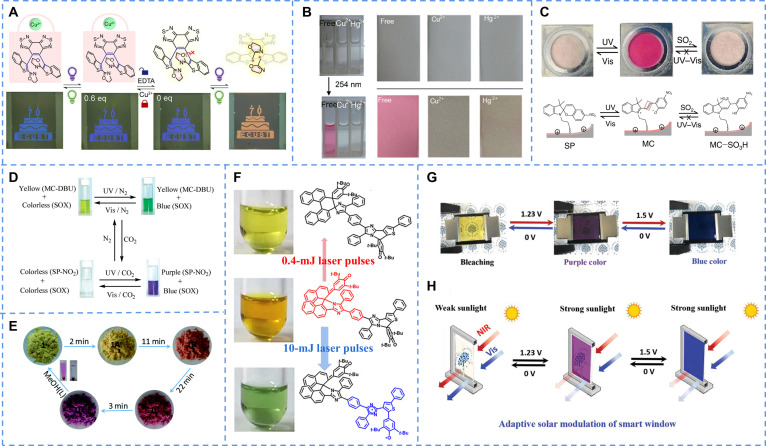
(A) The photoswitching ability of the copper-gated photoswitch molecule M-1o is effectively “locked” upon coordination with Cu^2+^ to form the 1o-Cu^2+^ complex and can only be restored after the removal of copper ions by adding the chelating agent EDTA. Reproduced with permission [133]. Copyright 2023, Elsevier Inc. (B) The photochromism of testing paper containing different ions. Reproduced with permission [134]. Copyright 2014, Royal Society of Chemistry. (C) The reversible molecular structure changes and corresponding colors among 3 forms under UV–Vis irradiation. Reproduced with permission [141]. Copyright 2023, Springer Nature. (D) The photo and N_2_ responsivity of the mixture of 1:1 SPNO_2_ and SOX in methanol with DBU. Reproduced with permission [142]. Copyright 2010, American Chemical Society. (E) The vapochromism along with time of [Cd(ipbp)Br]·1.75H_2_O under DEA vapor, which was reversible recover when the methanol solution was applied. Reproduced with permission [143]. Copyright 2017, Royal Society of Chemistry. (F) A schematic diagram of dual-color response regulated by visible light intensity: Weak light causes the system to fade from orange to yellow, while strong light changes the system to green. Reproduced with permission [146]. Copyright 2017, American Chemical Society. (G) Two single-electron reductions of PiPO-TTv. (H) Principles of functioning for smart windows constructed by PiPO-TTv material. Reproduced with permission [150]. Copyright 2022, Wiley-VCH GmbH.

The work by Zheng et al. [133] demonstrates an innovative copper ion-gated strategy, achieved by designing a photochromic diarylethene molecule featuring oxazoline functional groups, which enables multi-degree-of-freedom photoprogramming of soft helical microstructures (M-1o). Upon coordination with Cu^2+^ in solution, the molecule forms a stable complex, effectively “locking” its photoresponsiveness and exhibiting copper-gated photochromic behavior. The photoisomerization capability of the complex is nearly completely suppressed, and the photochromic performance is restored only after removing Cu^2+^ via the chelating agent EDTA. This approach not only allows precise tuning of the dynamic response range of reflection spectra by adjusting Cu^2+^ concentration but also achieves coordinated modulation of multiple optical degrees of freedom, including wavelength, tunable photonic band range, and fluorescence intensity, in liquid crystal helical microstructures. This pioneering work provides a novel regulatory approach for reconfigurable photonic devices and optical information processing, highlighting the great potential of ion-gated photochromic materials in complex environmentally adaptive optical systems and dynamic information encryption (Fig. 8A).

Zeng et al. [134] synthesized a novel phenanthrene bridging photochromic diarylethylene with double crown ether as the ion recognition group and studied the effects of different metal ions in solution (PBC). More than 10 metal ions were compared, and it was finally found that only Cu^2+^ and Hg^2+^ had a significant effect on the photochromic process, while other types did not change the photochromic behavior, indicating that the molecule was selective to only Cu^2+^ and Hg^2+^. When Cu^2+^ or Hg^2+^ was added, the red solution in the PSS state was bleached to colorless. The results showed that the ions combine with the crown ether, and the closed ring photochromic unit would be transformed into open form and changed color. This strategy for the visual detection of Cu^2+^ and Hg^2+^ facilitates the creation of low-cost, paper-based dry test strips (Fig. 8B). It presents a viable approach for rapid, on-site screening in areas such as environmental water analysis and food safety inspection, where expensive instrumentation is not accessible.

On the basis of the Lewis acid–base interaction between fluoride ions and boron atoms, the photochromic behavior of diarylethene derivatives can be effectively modulated. This specific and selective binding enables precise control over the photoresponse, facilitating the development of fluoride-gated photochromic materials (1O-B-Mes) [135]. Originally, it could change color to green under UV excitation. After the introduction of fluoride ions, fluoride adducts were formed, and the absorption spectrum was red-shifted, manifesting as the color changing from green to orange. The strong selectivity of the binding might be roughly attributed to 2 reasons: One is Lewis acid–base action, and the other is steric hindrance. It was easy for fluorine ions to combine with boron atoms due to their small steric resistance. This selective response transforms the system into a dual-function probe, capable of both the specific identification of fluoride ions and visual observation of microenvironmental changes.

Similarly, cyanide ions can be detected because of their high toxicity, making the development of rapid analytical techniques crucial. An ideal detection method should enable rapid and convenient analysis through a noncontact approach, and photochromic materials represent a promising candidate for achieving this goal. When cyanide ions were present in the system solution, they would combine with modified diarylethylene molecules to form adducts (1L-1o) [136]. However, in the presence of cyanide, its absorption spectrum developed a distinct peak at 406 nm, accompanied by a visible color shift to orange. More importantly, the system demonstrated a robust anti-interference capability against a wide range of competing anions. Even after more than 10 consecutive detection cycles, the material maintained a stable photochromic property. This robustness provides a solid foundation for its implementation in practical sensing devices and field-deployable detection cards.

As can be seen from the example above, ion-gated photochromic materials achieve precise control over photochromic processes by incorporating recognition units that interact specifically with target ions. These systems integrate ion-involved chemical recognition with photoresponsive behavior, thereby expanding the dimensionality of stimulus responses and enhancing the logical controllability and selectivity of the materials. Reported designs frequently use recognition motifs such as crown ethers and boronate esters, which enable highly selective and visual detection of ions including Cu^2+^, F^−^, and CN^−^. Such materials exhibit considerable potential for applications in environmental monitoring, biosensing, information encryption, and anticounterfeiting, offering innovative design pathways for the development of intelligent optically responsive materials and devices.

### Liquid-gated photochromism

The optical properties of certain photochromic molecules are profoundly influenced by their surrounding liquid environment. Solvent parameters such as polarity and electron-donating capacity can interact with the photochromic unit, thereby modulating its photoresponsive behavior and color output by regulating structural transformations or inducing adduct formation [137,138]. This inherent property of photochromic molecules enables the development of liquid-gated photochromic systems.

Among 3 photochromic coordination polymers prepared by Liu et al. [139] from NDI-based ligands and aromatic carboxylic acids, compound 1 (Zn-TPDC-1), which contains a diaryl system, exhibited excellent photochromic properties. Interestingly, when injected into different solvents, the polarity of the solvent would affect the color clearly. With the enhancement of the electron-supplying capacity of the solvent, a gradual red shift of the UV–Vis spectrum was observed, indicating that the solvent chromic effect was caused by the intermolecular electron transfer transition from the solvent to the DPMNI ligand in compound 1.

The metal–organic framework Eu(ipbp)_2_·1, constructed from a bipyridinium carboxylate ligand and europium ions, exhibits multistimulus-responsive chromic behavior, showing promise for applications in humidity sensing and optical anticounterfeiting [140]. This material demonstrates photochromism under visible light excitation and also displays a distinct color change upon interaction with water molecules. When exposed to ambient air, its structure incorporates water molecules to form coordinated water, resulting in a color transition from light yellow to light green. This process is reversible, as the original color is restored upon heating, which drives the desorption of the water molecules, enabling reversible color control.

### Gas-gated photochromism

In addition to their specific responsiveness to solvents, some photochromic molecules are also capable of recognizing gases. Gas-gated photochromism typically relies on specific interactions between gas molecules and the photochromic unit, often involving chemical reactions at active sites of the molecule. These interactions can alter the electronic structure or impede the photoisomerization pathway, thereby enabling gas-gated control over the photochromic switching behavior. Such materials show unique potential in applications such as highly selective gas sensing, environmental monitoring, and information encryption.

Zhang et al. [141] developed an inert/active state-switchable sensor and used the detection of SO_2_ as an example to investigate the light controlled on-demand recognition behavior of a SP/anodic aluminum oxide (AAO) nanochannel system (SP-AAO). The on-demand recognition of SO_2_ in the nanochannels was realized on the basis of the light-elicited isomerization of inert SP to activated MC, generating unsaturated (C═C), which was capable of binding to SO_2_ through nucleophilic addition to yield a new MC−SO_3_H adduct. SP was a light yellow solid, but the MC showed the purple and disappeared after SO_2_ treatment. This was attributed to the nucleophilic attack of SO_2_ toward (C═C) forming the MC−SO_3_H adduct, which broke the π-conjugated structure of MC (Fig. 8C). The high sensitivity and selectivity of these photochromic materials, coupled with their reversible optical switching and locking capabilities, make them highly promising for the development of next-generation intelligent nanosensors.

CO_2_ was an irritant in the photochromic process that could be easily removed or added to the system, so the detection of CO_2_ by SP was a new universal method [142]. This SP photochromic system regulated by CO_2_/DBU exhibits high biocompatibility and reversibility, holding significant potential for applications in multistate molecular logic gates and bioinspired microenvironment-responsive materials (Fig. 8D). The photochromic effect of SP was reversibly turned on and off by adding and removing CO_2_ from SP in an alcohol solution containing amidine (DBU) as a CO_2_ sensitizer (SP-DBU). When DBU, UV, visible, CO_2_, and N_2_ were introduced into each system, SP-NO_2_ and SP-1 (S-1 and S-6) in methanol exhibited similar behavior, enabling switching between the 4 color states (transparent, yellow, green, and blue).

Complexes formed by the combination of cadmium ions and bromopyridine not only had photochromic properties but could also respond characteristically to certain gases [143]. Through strategic modulation of solvent systems and counterions, they successfully synthesized 2 neutral cadmium-based MOFs (Cd-MOFs) featuring 2-dimensional layered structures. These MOFs exhibit pronounced photochromism, transitioning from light yellow to brown or green upon exposure to UV. More markedly, compound 1 demonstrates exceptional selectivity and rapid response as a vapochromic sensor for diethylamine (DEA), undergoing a distinct color change from light yellow to purplish-red within minutes at ambient temperature (Fig. 8E). These findings underscore the potential of these materials for applications in optoelectronic sensing and environmental monitoring, while also providing a new design strategy for developing advanced smart chromic materials.

### Intensity-gated photochromism

Intensity-gated photochromism has emerged as a significant research direction in the field of nonlinear photochemistry in recent years [144]. It is based on the stepwise 2-photon absorption process, enabling selective control of photochromic reactions through light intensity modulation [145]. Unlike conventional one-photon processes, such reactions rely on the nonlinear relationship between the lifetime of intermediate states and light intensity, thereby achieving intensity-selective responses even under low-power continuous-wave light or even sunlight.

Abe and colleagues [146] are central contributors to this research field. For example, they designed and synthesized a biphotochromic molecule constructed from a negative and a positive photochromic unit [Red-CF(1)]. The molecule ingeniously combines a visible-light-sensitive negative photochromic unit (BN-PIC) with a positive photochromic unit (TPIC) that is originally insensitive to visible light. The key to its design is that simply by adjusting the intensity of 470-nm visible light, the reaction pathway can be reversibly switched: Under weak light, the reaction of BN-PIC dominates, yielding a yellow product with slower recovery; under strong light, TPIC is excited via energy transfer, producing a green product with faster recovery (Fig. 8F). This work not only provides a novel molecular design paradigm for developing intelligent photoswitchable systems capable of multicolor output controlled solely by visible light intensity but also demonstrates promising application prospects in fields such as multilevel information encryption, dynamic optical anticounterfeiting, and intensity-gated logic devices.

DASAs exhibit unique performance in the field of time-resolved asymmetric encryption systems. By precisely controlling the incident light intensity, the photochromic kinetics of DASA molecules in polymer matrices can be effectively regulated (DASA-PCL) [147]. Research demonstrates that dynamic control can be achieved by adjusting the irradiation light intensity: Higher light intensity accelerates fading, while lower light intensity significantly delays the process. Utilizing this light-intensity modulation mechanism, researchers have realized sequential display and concealment of multilayered information along the time dimension. This system enables the time-resolved display of encrypted information using common light sources at different intensities. Only recipients who simultaneously possess the correct light intensity and reading sequence can extract the genuine content, thereby demonstrating promising application potential in anticounterfeiting labels and dynamic information encryption.

In summary, ion-, liquid-, gas-, and intensity-gated photochromic materials, as significant branches of unconventional multigated systems, demonstrate considerable potential in specific application scenarios owing to their unique response mechanisms. The in-depth exploration of these unconventional gated systems not only enriches the design repertoire of multigated photochromic materials but also indicates their pivotal and irreplaceable role in future applications such as highly selective chemical sensing, microenvironment visualization, and adaptive intelligent devices.

## Multiresponse Photochromism

On the basis of the foregoing discussion, which has centered on various intelligent response systems constructed by combining photochromism with individual external stimuli such as protons, electric current, temperature, and mechanical force, it is evident that these systems each possess distinct characteristics. However, for future highly integrated intelligent applications, the development of advanced materials capable of responding to multiple environmental signals in parallel has become an imperative. It is precisely within this context that this section will focus on a novel class of multiresponsive photochromic materials.

For example, Tong et al. [148] developed a new photochromic system based on rhodamine B salicylaldehyde hydrazone metal complex, in which the photochromic behavior can be precisely modulated by multiple factors including light, specific metal ions, solvent polarity, molecular substituents, and temperature. UV light promoted the isomerization of the salicylaldehyde hydrazone moiety from the enol form to the keto form and subsequently induced the spirolactam ring opening in the rhodamine B part and caused the photochromic reaction. Upon 365-nm irradiation, the light yellow solution underwent a rapid color evolution to purple. Moreover, the recovery rate of the photochromic reaction is intricately governed by the identity of the coordinated metal ion. This modulation arises from the distinct coordination environments and binding strengths provided by different metals, resulting in the fastest recovery for zinc complexes (ZnRho) and the slowest for their copper counterparts. Furthermore, specific conditions such as solvent polarity, substituent structure, and temperature also influence both the photocoloration and recovery processes. The ability to precisely program the recovery kinetics through metal selection may open a pathway for developing intelligent photochromic platforms for sensing and anticounterfeiting.

On the basis of the progress already achieved in thermochromic smart windows [149], more intelligent materials with multistimulus-responsive capabilities (including light, heat, and electricity) introduce new dimensions to the field. Kim et al. [150] successfully constructed the novel PiPO-TTv material based on a new strategy for the synthesis of the multistimulus-responsive single polymeric material, which had different applications. The new material was mainly a polymer formed by thiazole violet, which was a common photochromic and electrochromic material. As the voltage increased, the color changed obviously (Fig. 8G and H). The PiPO-TTv redox reaction mechanism showed that the first electron transfer produced free radicals, turning the device purple, while the second electron transfer turned the molecule into a fully reduced state, changing its hue from purple to blue. Then, solution was prepared and evenly coated in the temperature control sensor device to realize the intelligent sensing window. When the temperature rose, the originally transparent glass gradually became blurred. After the temperature was restored, it became transparent again, which promoted the development of efficient equipment. Such superior environmental adaptability and tunability—significantly boosting the material’s controllability and versatility—pave the way for its unprecedented application potential in future such as intelligent households.

In multiresponsive photochromic materials, the simultaneous integration and precise regulation of synergistic responses to multiple stimuli such as light, ions, and temperature remain a central challenge for future development. Through rational molecular design and the construction of composite systems, these materials hold the potential to enable revolutionary applications in intelligent sensors, adaptive optical devices, and biomedicine, advancing materials science toward a higher dimension of intelligence and integration.

## Conclusion and Outlook

### Summary

Multigated photochromic materials represent a significant advancement in the field of light-responsive smart materials, achieved through the strategic integration of photochromic units with diverse external stimulus-responsive modules (such as pH, electric fields, mechanical force, and temperature). This integration enables precise control over the photoisomerization process. In this review, we systematically summarize the design strategies and fundamental mechanisms underlying various gating approaches, as well as their emerging applications in information encryption, intelligent sensing, and logic operations. To provide a clear and comparative overview of these diverse gating modalities, we have specifically compiled their key characteristics into a concise table (Table 2). These developments highlight the key advantage of multigated systems over conventional photochromic materials: the ability to construct sophisticated stimulus–response logic, thereby endowing materials with enhanced environmental intelligence and functional programmability.

**Table 2.** Comparison of gating types, mechanisms, and applications for multigated photochromic materials

Number	Gating Response Type	Primary modulation method	Main mechanism	Advantages	Disadvantages	Future applications
1	pH-gated	Proton (H^+^) concentration modulation	Protonation induces structural changes	Fast response, good reversibility, high utility in biological	Sensitive to pH range, limited cycling stability	Biosensors, environmental pH monitoring, information encryption, smart-responsive surfaces
2	Electro-gated	Voltage/current	Redox reactions trigger structural transformations	Fast response speed, high control precision, integration with electronic devices	Electrode contact required, relatively high cost, challenging for large-scale fabrication	Smart displays, electrochromic windows, molecular logic gates, wearable electronics
3	Mechano-gated	Grinding, stretching, ultrasonication	Chemical bond cleavage or alteration of molecular arrangement	Solid-state response, solvent-free, suitable for stress visualization	Complex stress–response relationship, prone to material fatigue	Stress-sensing coatings, rewritable optical storage, smart textiles
4	Temperature-gated	Heating/cooling	Thermally induced structural changes	Noncontact control, suitable for temperature-sensitive scenarios	Slow response speed, narrow operational temperature range, difficulty adapting to harsh environments	Smart thermochromic windows, temperature sensors, advanced anticounterfeiting labels
5	Wavelength-gated	Different light sources (x-ray, Vis, NIR)	Wavelength-specific excitation matching molecular energy levels	Excellent wavelength selectivity, multicolor output	Strong dependence, complex system design	Multilevel information encryption, bioimaging and therapy, radiation detection, reconfigurable photonic devices
6	Ion-gated	Specific ion recognition/binding	Ion coordination/bonding to recognition units alters electronic structure	High selectivity, visual detection capability	Susceptible to ion interference, complex synthesis of recognition motifs	Ion sensors, environmental monitoring, biochemical detection, logic gates and information processing
7	Liquid-gated	Solvent polarity	Solvent–molecule interactions modulate electron transfer	Diverse responses, solvent recognition	Dependent on solvent environment, limited solid-state application	Solvent sensors, chemical analysis, microenvironment probing, multistate displays
8	Gas-gated	Gas molecule adsorption/reaction	Interaction between gas molecules and photochromic units	High sensitivity, rapid response	Limited gas selectivity, possible humidity interference	Gas sensors, environmental monitoring
9	Intensity-gated	Light intensity modulation	Nonlinear optical effects	Depth-selective excitation, 3-dimensional lithography and bioimaging	High-intensity light sources, high system cost, complex material design	3-dimensional optical data storage, 2-photon microscopy, intensity-dependent logic switches

### Current challenges

Despite considerable progress in this field, the transition of multigated photochromic materials from conceptual design to practical implementation faces several critical challenges. First, the structural diversity of molecular systems requires significant expansion, as current research predominantly focuses on derivatives of a limited number of classical photochromic scaffolds, with insufficient development of novel, intrinsically multiresponsive molecular platforms. Second, the stability and fatigue resistance remain fundamental bottlenecks for practical applications, particularly in complex or harsh environments where the spectral performance and cycling durability of materials often fall short of operational requirements. Furthermore, issues such as interference between multiple stimuli in systems, limited efficiency, and response rates, along with difficulties in processability and scalable fabrication, collectively constitute major obstacles to their industrial adoption.

### Future application directions

Looking forward, overcoming the challenges in molecular diversity, stability, and processability of multigated photochromic materials requires coordinated research strategies to fully realize their application potential. In the biomedical field, for instance, developing novel photochromism molecular architectures with enhanced biocompatibility and stability is essential to construct systems that respond cooperatively to NIR light and specific biomarkers, enabling precise diagnostics and targeted therapies. For energy and building applications, efforts should focus on improving environmental resilience and cycling ability to create light/thermal/electric multiresponsive smart windows capable of dynamic solar energy management, thereby offering innovative solutions for building energy efficiency. In information technology, molecular engineering approaches must optimize switching speed and fatigue resistance to establish a foundation for reconfigurable photonic devices, ultrahigh-density optical storage, and advanced anticounterfeiting technologies. Furthermore, integration with flexible electronics will facilitate the development of interactive wearable devices that enable real-time visualization of mechanical strain and optical signals. Ultimately, through interdisciplinary collaboration—particularly in advancing molecular design, enhancing material stability, and integrating artificial intelligence with advanced manufacturing—multigated photochromic materials are poised to serve as key enablers for next-generation adaptive intelligent systems.
